# Une cause inattendue de présentation de front: l’iniencéphalie

**DOI:** 10.11604/pamj.2016.25.188.10900

**Published:** 2016-11-24

**Authors:** Amira Ayachi, Mechaal Mourali

**Affiliations:** 1 Service de Gynécologie et Obstétrique, CHU Bougatfa, Bizerte, Tunisie; 2 Faculté de Médecine de Tunis, Université Tunis El Manar, Tunisie

**Keywords:** Iniencephaly, prenatal diagnosis, ultrasound, Iniencephaly, prenatal diagnosis, ultrasound

## Image en médecine

Il s'agit d'une patiente G2P2, admise en travail à un terme de 38 SA+3 jrs. L'examen trouve une patient à dilatation complète, poche des eaux rompues, et une présentation de front fixée. L'enregistrement du rythme cardiaque fœtal était interprété comme normal. La patiente a été césarisée pour une présentation de front. L'examen en salle de naissance trouve un nouveau-né de sexe féminin, PN= 3Kg700, en détresse respiratoire, l'intubation n'a pas été possible du fait de l'hyperextension (A). Une radiographie a été réalisée montrant une malformation thoraco-vertébrale dorsale impossibilité de spécifier les vertèbres cervicales et une cyphose rachidienne dorsale (B). Le nouveau-né est décédé à H5 de vie.

**Figure 1:**
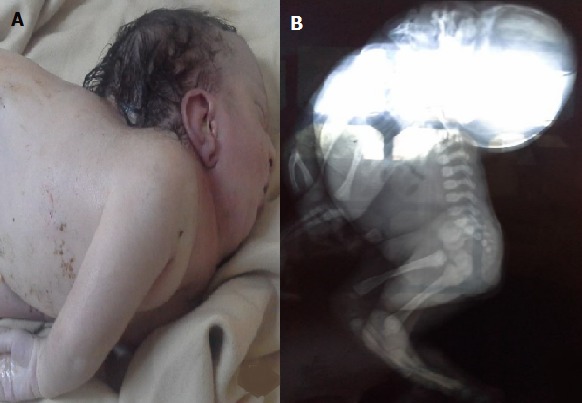
Aspect du nouveau-né avec iniencéphalie; A): tête en hyperextension; B) : radiographie montrant la malformation thoraco-vertébrale, la cyphose rachidienne dorsale et la non visualisation des vertèbres cervicales

